# A comparison between passive and active case finding in TB control in the Arkhangelsk region

**DOI:** 10.3402/ijch.v73.23515

**Published:** 2014-02-14

**Authors:** Vladimir N. Kuznetsov, Andrej M. Grjibovski, Andrey O. Mariandyshev, Eva Johansson, Gunnar A. Bjune

**Affiliations:** 1 Institute of Health and Society, University of Oslo, Oslo, Norway; 2 Institute of Mental Medicine, Northern State Medical University, Arkhangelsk, Russia; 3 International School of Public Health, Northern State Medical University, Arkhangelsk, Russia; 4 Department of International Public Health, Norwegian Institute of Public Health, Oslo, Norway; 5 Department of Tuberculosis, Northern State Medical University, Arkhangelsk, Russia; 6 Karolinska Institute, Stockholm, Sweden; 7 Nordic School of Public Health, Gothenburg, Sweden

**Keywords:** tuberculosis, diagnostic delay, active and passive case finding

## Abstract

**Background:**

In Russia, active case finding (ACF) for certain population groups has been practiced uninterruptedly for many decades, but no studies comparing ACF and passive case finding (PCF) approaches in Russia have been published.

**Objective:**

The aim of this study was to describe the main differences in symptoms and diagnostic delay between patients who come to TB services through PCF and ACF strategies.

**Methods:**

A cross-sectional study was conducted among 453 new pulmonary tuberculosis (PTB) patients, who met criteria of TB diagnostic delay in Arkhangelsk.

**Results:**

ACF patients used self-treatment more often than PCF patients (90.1% vs. 24.6%) and 36.3% of them were alcohol abusers (as opposed to only 26.2% of PCF patients). The median patient delay (PD) in PCF was 4 weeks, IQR (1–8 weeks), and less than 1 week in ACF. Twenty-three per cent of the PCF patients were seen by a medical provider within the first week of their illness onset.

**Conclusion:**

Patients diagnosed through ACF tended to under-report their TB symptoms and showed low attention to their own health. However, ACF allowed for discovering TB patients earlier than PCF, and this was also the case for alcohol abusing patients. PCF systems should be supplemented with ACF strategies.

Since the 1990s there has been a substantial improvement in access to high-quality tuberculosis (TB) services globally (1). However, too many people still remain undiagnosed or diagnosed late. This leads to a persistent high risk of transmission in some societies. TB screening can reduce the delay in diagnosis and thus the risk of poor disease outcomes and TB transmission (2).

In 2013, the World Health Organization (WHO) issued guidelines for active TB screening defined as “the systematic identification of people with suspected active TB, in a predetermined target group, using tests, examinations or other procedures that can be applied rapidly” (2). The aim of screening is to efficiently distinguish people with a high probability of having active TB from those with low probability (2). Health providers initiate systematic screening of people who, for some reason, do not seek health care; people have no symptoms or cannot recognize them; people who consider their health problem to not warrant medical attention; or those who face barriers to accessing health care (2).

National TB control programs might have low impact on true incidence because patients are diagnosed and treated too late (3). Active case finding (ACF) could be effective in providing early diagnosis and is typically aimed at TB high-risk groups (homeless, prisoners, patients in nursing homes, and residents of impoverished areas). Because ACF has to examine people at certain intervals, some patients might become infectious between the regular screenings and delay their health seeking because they depend on the regular screenings. The effects of a successful ACF program would thus be optimal if passive case finding (PCF) is strengthened (4). To be successful, ACF should be provided with the expansion of TB diagnostic facilities in the community (5) and while promoting favourable health seeking behaviours (6).

The DOTS strategy is based entirely on PCF and it is due time to consider whether ACF could strengthen it (7). Cost-efficiency of ACF depends on whether it reduces secondary cases in the community (8), prevents deaths, saves life years and increases quality-adjusted life years (9). Additionally, older, female and HIV-infected patients with TB are more difficult to reach by PCF (10).

Most patients are automatically delayed because the definition of suspected TB requires a cough for more than 2 weeks duration. Adding to that is the patient delay (PD) in seeking care and the health systems delay (HSD) in reaching the correct diagnosis and initiating adequate TB treatment. Some pulmonary tuberculosis (PTB) cases may be lost because a cough is common for smokers and is considered a “normal” thing. For example, in Sri Lanka a cough of more than 2 weeks duration had been ignored in many of the TB cases (11, 12). Moreover, respiratory symptoms are often missed by doctors during examination as well as the production of sputum (12). So, patients may circulate as “non-tuberculosis” in the health system for a long time. Commonly, all patients having cough, fever and shortness of breath are treated as having simple respiratory tract infection. Only when the patient does not respond to treatment is PTB considered as a “second line” possibility. The low sensitivity and specificity of the symptoms, signs and the general examination of TB cause health system delay (HSD) (13, 14). Sputum culture-negative individuals with chest X-ray abnormalities are at a high risk of disease progression if they are undiagnosed as TB (15). PTB should be suspected and excluded at least by chest X-ray, which is readily available technology (11).

Alcohol problems have been associated with TB disease and diagnostic delay (DD) (16–21) caused by decreasing social activity. These individuals face employment difficulties, homelessness, social marginalization, increased risk of infection, re-infection and co-infection with HIV (19–23). At population level we have described an association between the incidence of alcohol psychoses and the TB incidence in the same year in northern Russia suggesting a link between excessive levels of alcohol consumption and TB incidence (24).

So, the population suffering from TB is not representative of the “usual” patients in outpatient clinics; they rarely seek care, and when they seek help they are less likely to be diagnosed. PCF strategy supposed that patients are generally “active care seekers” who are examined for TB cases because they have symptoms compatible with TB. The weakest point of PCF is “waiting for patients” willingness to ask for help (25, 26). Many individuals with smear-negative bacteriologically positive pulmonary TB do not report any symptoms at all (27–29). In South Korea, the prevalence rate of pulmonary TB among homeless people was much higher than among the general population (30). The PCF approach may leave a large pool of undetected prevalent TB cases even in settings with well-functioning TB programs (31).

In Russia, ACF for certain population groups has been practiced uninterruptedly for many decades. But no studies comparing ACF and PCF approaches in Russia have been published. The aim of this study was to describe the main differences in symptoms and DD between patients who come to TB services through PCF and ACF strategies.

## Methods

### Setting

The Arkhangelsk region is located 1,200 km north of Moscow. Its total area is 589,913 km^2^. The population, as of 2012, was 1,213,533 people. The region consists of 20 districts and includes 15 cities and more than 4,000 villages (32–34).

### TB management in the Arkhangelsk region

Each district has a TB specialist. Arkhangelsk Regional Anti-tuberculosis Dispensary (ARAD) is the main institution responsible for the care of TB patients. It has both inpatient and outpatient departments and can provide treatment for all cases of TB, including multidrug-resistant TB (MDR TB) and extensively drug-resistant TB (XDR TB) cases.

All smear-positive cases are hospitalized at ARAD, and smear-negative patients start the outpatient treatment. All examinations are free of charge, but the travel costs rest on the patient. All patients with TB, as well as all TB deaths, in the Arkhangelsk region are registered in ARAD. An extensive questionnaire including socio-demographics, symptoms and delay is filled in for each diagnosed patient. No private practitioners treat TB.

In spite of this, DOTS has been provided in the region since 1998. The MDR TB management program dates from 2005, and active screening for TB is conducted according to Russian regulations (35–37). The WHO case definition is used here (38).

All children younger than 14 years are tested by tuberculin skin test annually. Military, maternity staff, household contacts with tubercular people and ex-TB patients after cure or completion of treatment are tested once every 2 years; HIV-infected people, mentally ill people, and people released from prison are examined every 2 years; and prisoners and people at pre-trial centres are examined by X-ray twice a year. All staff working in any children's or social services (including school and preschool teachers), as well as patients with any chronic lung disease, any gastrointestinal disease, any chronic urinary diseases, diabetes, as well as homeless people and migrants must be examined once a year (including X-ray examination). People with any respiratory symptoms who enter the hospital for reasons other than TB, individuals living with pregnant women and newborns, military recruits, persons with first diagnosis of HIV and people who work with hazardous and harmful substances must undergo an initial examination. Other populations older than 14 years should be examined once every 2 years (35–37).

In 2012, 486,437 people were examined by X-ray digital screening for diagnostic tuberculosis (ACF). This constituted 49.4% of the entire adult population of the region. In 2012, 12,428 patients suspected of having TB, who had a cough lasting more than 2 weeks were asked to submit to a sputum test by the microscopy method (PCF) and 181 TB cases were diagnosed (1.46% positive diagnostic). Among 431 new TB cases, 181 (41.9%) were diagnosed by PCF; 198 (45.5%) by X-ray examination (ACF) (34 (7.9%) because of contact with TB patients; 18 (4.2%) by autopsy); and 2 (0.5%) by the tuberculin skin test in children. (Report of regional TB service). Among new cases, 66 ACF patients (33.3%) and 110 PCF patients (61%) had positive smear microscopy (Figure 3).

Before initiation of the treatment, every TB patient is examined by the smear microscopy, GeneXpert, Bactec-MGIT, Lowenstein Jensen media, and digital X-ray and tomography X-ray examination. Smear and culture positive patients are examined for drug susceptible test mycobacteria by the LPA method.

**Fig. 1:**
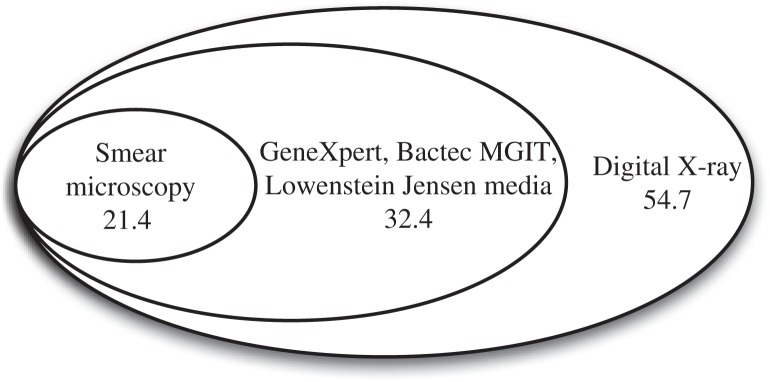
Number of new and relapsed TB cases (per 100,000) confirmed by various methods in the Arkhangelsk region 2012. Note: The cases in a bigger ellipsis include smaller ones (Report of regional TB service).

**Fig. 2:**
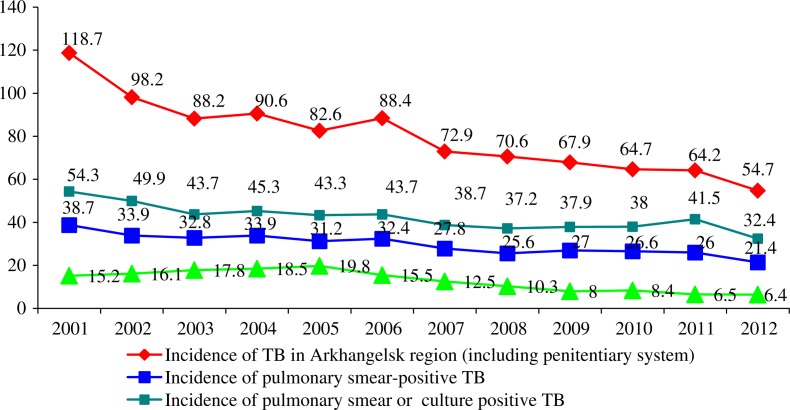
TB Incidence including penitentiary system (new cases and relapses) and mortality in Arkhangelsk Region 2001–2012 (per 100,000 population).

In 2012, 239 (21.4/100,000) new and relapsed TB cases were confirmed by smear microscopy, 362 (32.4/100,000) cases by smear microscopy and GeneXpert, Bactec MGIT, Lowenstein Jensen media and 611 (54.7/100,000) by all these methods plus X-ray examination only (Figures 1 and 2). TB patients with X-ray abnormalities without bacteriological confirmation received 2 courses of antibiotic therapy before registration of their TB cases.

**Fig. 3:**
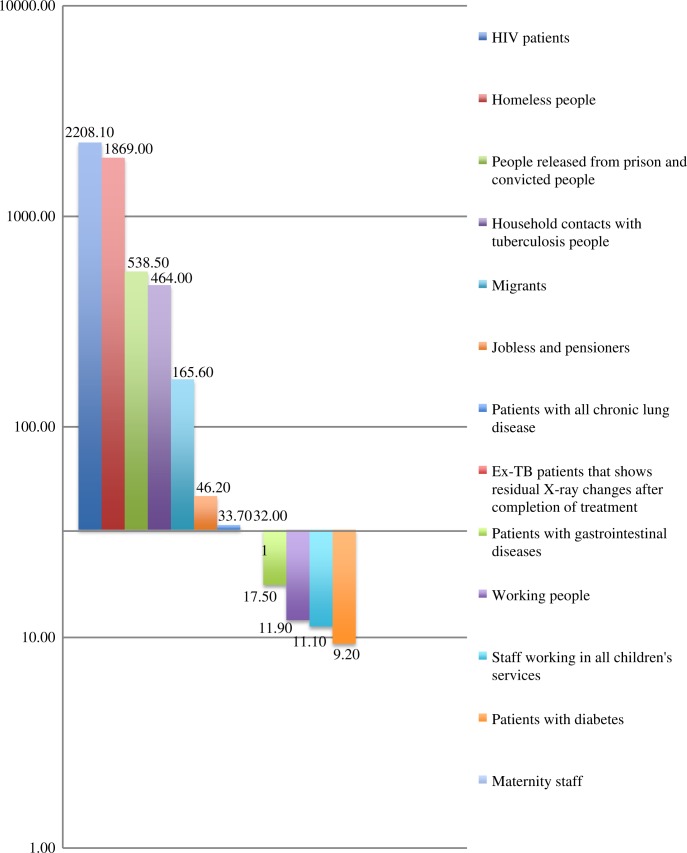
Number of TB cases revealed by ACF (per 100,000 of the population). The TB incidence confirmed bacteriology in the Arkhangelsk region 2012 (32.4 per 100,000 citizens) was taken as a cut-off line.

Among 1,213,533 citizens in the civil sector excluding the penitentiary system there were diagnosed 431 new TB cases, 82 relapses, 7 treatment after loss to follow up and 5 treatment after failure patients. Among them were 155 new MDR TB patients (37.5%) and 7 patients with XDR TB. Among the patients, 68.2% of new cases experienced treatment success, 11.6% continued MDR-TB treatment, 46.2% of relapses experienced treatment success and 22.0% were continued MDR-TB treatment patients, 71.4% treatment loss to follow up patients and, 60.0% of failure patients were cured and 1 patient continued MDR-TB treatment. Total treatment success (cured and treatment completed) among all TB patients registered in 2012 was 63.5% and continued MDR-TB treatment was 13.1% (Figure 3).

### Definition of variables

There is no definite time for DD in literature and no WHO guidelines for this. When comparing DD for patients diagnosed with TB by ACF versus PCF we decided to select the patients who had positive smear microscopy because these patients are more infectious in society. The contribution of ACF to reduce community transmission might be substantial in a situation where half of the population is subject to regular screening (35–37). We used the following classification of DD: patient delay (PD) is the duration of time from onset of symptoms to the first interaction with a medical health provider, health system delay (HSD) is the time from the first visit to the initiation of treatment and the total delay (TD) is the time from onset of symptoms to initiation of treatment (39–41). PCF was defined as detecting active TB disease among symptomatic patients who contact the health care system for diagnosis of symptoms (42) and ACF as a health care system special effort to increase the detection of TB in a given population (43).

### Sample and design

A cross-sectional study was conducted between 1 March 2012 and 5 February 2013 among new smear-positive PTB patients in the Arkhangelsk region. We used continuous sampling for data gathering and included all patients who met criteria of TB DD (n = 453) during 2007–2011. We did not conduct face-to-face interviews; all the data were taken from individual medical records.

The major pulmonary symptoms included were presence of cough, production of sputum, weakness, fever, dyspnoea, loss of appetite, and sweating. Alcohol and smoking anamnesis were also included. The group was split into 2 groups: patients who came on their own to the health system because of symptoms (PCF) and those who came for the prescribed regular medical examination (ACF). Differences in symptoms and delays in the subgroups were estimated.

### Statistical analysis

SPSS version 20.0 was used for analysis. Because the data were skewed, intra-group differences were compared using the non-parametric Mann–Whitney criterion for 2 independent samples, Kruskal–Wallis tests for more than 2 independent samples in ordinal and categorical data. Medians, modes and interquartile scores were calculated. Linear regression analyses were performed to assess the relative impact of predictor variables on the outcome variables, and a *p*-value of <0.05 was considered statistically significant. We used *p*-value of <0.017 for data evaluation in pairwise comparison with 3 variables using Mann–Whitney criterion.

We included time from initial symptoms till TB diagnosis (PD) and time from first visit to the health system till TB treatment initiation (HSD) and time from symptoms onset till treatment initiation (TD) as the dependent variables and various patient characteristics as the independent variables.

## Results

### Patient characteristics

The patients were new smear-positive PTB patients (81.5% males and 18.5% females), aged 45.3 years (range being 20–86 years). Socio-economic characters of the patients are presented in Table I. Most of the patients lived in Arkhangelsk, were jobless and had secondary school education. Sixty-six per cent of patients in the PCF group and 63.3% of the patients in the ACF group lived alone. Thirty per cent of the patients had been imprisoned; 90% used alcohol, and half of them reported dependency; more than 80% were smokers and the mean time of smoking was 24.0 years (IQR 16–30). Only 3% had ever used psychoactive drugs. Eighty per cent of the patients were unemployed and 11% of them had no housing (Tables I and IV).

**Table I:** Socio-economic characteristics of patients with TB DD

Characteristics	Number
Family status	
Single	210
Divorced	30
Widow(er)	60
Civil marriage	61
Married	91
Education	
High	119
Middle	254
Basic	80
Living conditions	
Good	186
Poor	217
No accommodation	50
Work	
Permanent	74
Periodic	56
Retired	27
Disabled	28
Unemployed	258
Alcohol	
No	47
User	270
Abuser	136
Reason for visit to health system	
Annual examination	157
Symptoms	282
Contact	14
First medical institution	
Ambulance	95
Doctor assistant	22
District hospital	311
Anti-TB dispensary	25
Self-treatment	
No	401
Yes	52

### Symptoms and health-seeking period

During the first visit to health care, 65.3% of patients reported the following symptoms: cough (90.0%), fever (64.7%), weight loss (55.4%), sputum (40.2%), weakness (33.6%), sweating (26.9%), dyspnoea (25.8%) and loss of appetite (25.8%). However, only 62.3% of the patients came to the health care system because of symptoms (PCF). Others were called for a prescribed medical examination and digital X-ray screening (ACF).

One fifth of the patients (21.0%) came to a hospital as an emergency. All patients had visited a medical health provider initially (68.7% of them came to a district health centre). Most of them (88.5%) did not use self-treatment before visiting the health centre.

Most of the patients (77.5%) had a HSD of less than 2 weeks (Tables II and III). The median PD was 4 weeks, IQR (1–8 weeks). Based on the cumulative distribution 44.2% of the subjects consulted a medical health provider within the first week of the onset of their illness. The longest delay was reported to be 192 weeks.

**Table II:** Parameters of DDs (weeks)

	PD			HSD		
		
Variables	Mean	Median (Q_1_–Q_3_)	*p*	Mean	Median (Q_1_–Q_3_)	*p*
Sex						
Male	5.07	2 (0–6)	0.07	1.34	0 (0–0)	0.756
Female	3.05	0.5 (0–4)		1.06	0 (0–0)	
Family						
Single	4.47	1.0 (0–5)	0.71	1.42	0 (0–0)	0.11
Married	5.14	1 (0–6)		1.05	0 (0–1)	
Education						
High	4.71	3 (0–6)	0.139	1.84	0 (0–2)	0.07
Middle	4.59	1 (0–4)		0.91	0 (0–0)	
Basic	5.00	1 (0–4)		1.69	0 (0–0)	
Prison						
No	4.45	2 (0–6)	0.23	1.11	0 (0–0)	0.55
Yes	5.25	1 (0–4)		1.71	0 (0–1)	
Housing						
None/poor	4.06	1 (0–4)	0.13	1.09	0 (0–0)	0.24
Yes	5.61	2 (0–6)		1.58	0 (0–0)	
Job						
Work	3.44	1 (0–6)	0.72	1.13	0 (0–0)	0.29
Jobless	5.05	1 (0–6)		1.34	0 (0–0)	
Alcohol						
No	3.28	2 (0–5)	0.016	2.13	0 (0–2)	0.017
Use	5.46	2 (0–6.25)		1.41	0 (0–0)	
Abuse	3.66	0 (0–4)		0.76	0 (0–0)	
Reason for seeking medical help						
ACF	1.01	0 (0–0)	0.000	1.02	0 (0–0)	0.19
PCF	6.93	4 (1–8)		1.45	0 (0–0)	
Self-treatment						
No	4.44	1 (0–4)	0.000	1.31	0 (0–0)	0.007
Yes	6.67	4 (3–8)		1.13	0 (0–1)	
First medical institution						
Ambulance	4.14	1 (1–4)	0.024	0.95	0 (0–0)	0.629
Doctor assistant	3.91	2 (0–6)		1.05	0 (0–2.25)	
District health centre	5.18	2 (0–6)		1.51	0 (0–0)	
Anti-TB dispensary	1.44	0 (0–2)		0.12	0 (0–0)	

**Table III:** Parameters of DDs for patients who had symptoms (weeks)

	PCF											
		
	PD			HSD			ACF with symptoms					
			
Variables	Mean	Median (Q_1_–Q_3_)	*p*	Mean	Median (Q_1_–Q_3_)	*p*	Mean	Median (Q_1_–Q_3_)	*p*	Mean	Median (Q_1_–Q_3_)	*P*
Sex												
Male	7.32	4 (1–8)	1.94	1.39	0 (0–0)	0.39	2.03	0 (0–4)	–	1.56	0 (0–1.25)	0.25
Female	4.91	2 (0.75–8)		1.78	0 (0–1.25)		3.75	2.5 (1.25–5.5)		0.63	0 (0–1.75)	
Family												
Single	6.58	4 (1–8)	0.78	1.68	0 (0–0)	0.11	2.47	2 (0–4)	0.72	1.06	0 (0–0)	0.36
Married	7.64	3 (1–8)		0.98	0 (0–1)		2.0	0.5 (0–4.5)		2.4	1.5 (0–4)	
Education												
High or Middle	6.80	4 (1–8)	0.3	1.22	0 (0–1)	0.65	2.36	1 (0–4)	0.84	1.49	0 (0–2)	0.18
Basic	7.52	3 (0–7.25)		2.56	0 (0–0)		2.33	1 (0–1)		0	0 (0–0)	
Housing												
Good	8.39	4 (1–8)	0.04	1.39	0 (0–1)	0.45	2	0 (0–3)	–	0.4	0 (0–0.5)	0.03
None/poor	5.86	3 (0–8)		1.5	0 (0–0)		2.6	2 (0–4)		0.48	0 (0–1)	
Job												
Yes	5.27	4 (1–7)	0.83	0.97	0 (0–0)	0.90	2.38	1.5 (0.25–3.75)	0.6	2.38	1 (0–3.5)	0.043
Jobless	7.36	4 (1–8)		1.58	0 (0–1)		2.35	0.5 (0–4)		1.15	0 (0–0.25)	
Alcohol												
No	4.90	4 (2–7)	0.70	2.29	0 (0–2)	0.25	0.4	0 (0–1)	0.6	2.38	0 (0–5.5)	0.53
Use	7.18	4 (1–8)		1.35	0 (0–0)		2.62	2 (0–4)		1.27	0 (0–1.5)	
Self-treatment												
No	6.79	3 (0–8)	0.001	1.48	0 (0–0)	0.44	2.26	0 (0–4)	0.18	1.51	0 (0–1)	0.44
Yes	7.71	6 (3–10)		1.33	0 (0–1.25)		2.86	3 (1–4)		0	0 (0–2)	

Patients who used alcohol had a longer PD than those who did not (*p*=0.015); patients who used self-treatment also had a longer time of PD (Table III). Patients who had a high level of education had longer HSD than those who only graduated from primary school (*p*=0.006). In a linear regression analysis the symptoms correlated with time from the first visit to contact TB service (β=6.081; 95% CI 3.83, 8.33; R^2^=0.059).

### Active case finding versus passive case finding

There were 171 (37.8%) patients diagnosed by ACF and 282 by PCF. Patients who came by ACF had less time from the symptoms onset until TB diagnosis (1.0 weeks vs. 6.9 weeks, *p*<0.000) and less time TD (1.0 vs. 1.5, *p*<0.000) than those who had come by PCF. Forty-two patients in the ACF group had symptoms before first contact with the health system and showed mean PD=2.36 weeks (Mo=0; IQR 0–4) and mean TD=3.1 (Mo=0, IQR 0–3). Those who had inadequate or no housing and had employment showed longer HSD time Patients in ACF group used self-treatment more often than PCF patients (90.1% vs. 24.6%; *p*=0.002). Additionally, 36.3% of ACF patients were alcohol abusers while only 26.2% of PCF patients abused alcohol (*p*=0.016). Among PCF patients the median PD was 4 weeks, IQR (1–8 weeks), while the median time for ACF was less than 1 week.

Twenty-three per cent of the PCF patients were seen by a medical provider within the first week of their illness onset (Table III). In linear regression analysis, symptoms correlated with time from first visit to contact TB service (β= 6.143; 95% CI 0.52, 11.77; R^2^=0.016) (Table IV).

**Table IV:** Time of patients’ and health system delay in some countries

Country	PD	HSD	Reference
Vietnam	3 weeks	1 week	(44)
China	10 days	2 days	(45)
Ethiopia	30 days	21 days	(46)
Nepal	50 days	18 days	(47)
France	47 days	48 days	(48)
Spain	–	6 days	(41)
Uganda	1 week	9 weeks	(49)
Norway	28 days	33 days	(50)
Rwanda	25 days	28 days	(51)
Ethiopia	60 days	6 days	(52)
South Africa	30 days	14 days	(53)
Russia	16,7 days	50 days	(54)

## Discussion

We described the symptoms of patients who contacted the TB services through ACF and PCF strategies. We gathered all data about all patients in the Arkhangelsk region with DD for 5 years, so we described a common pattern of reporting patients’ complains. There is no missing data, but the quality of anamnesis in the medical documentation is doubtful.

In 2012, 12,428 patients suspected of having TB were asked to submit to a sputum test (PCF), which revealed 181 TB cases (14.9/100,000). Among 431 new TB cases, 41.9% were diagnosed by PCF and 58.1% by ACF (Report of regional TB service). The TB DD is an indicator of work efficiency at outpatient clinics, and family doctors have financial responsibility for timely diagnosis, so, these data are valid. Each case of TB death or a severe case of TB is discussed at the medical conference. Administrators of outpatient clinics control the quality of diagnosis of socially important diseases (oncological diseases, TB, HIV and others). Clinic administrators can reduce a doctor's monthly salary if the findings of the medical conference confirm poor performance.

ACF allowed identifying TB patients within the first week, while patients who arrived at the health care service by PCF had 6.9 weeks from symptoms onset until contact with the health system. Patients in the ACF group rarely had TB symptoms and used self-treatment more often than PCF patients. A possible explanation is that self-treatment decreased the intensity of the TB symptoms, which reduced the desire of people to contact the health system.

Additionally, 36.3% of ACF patients were alcohol abusers versus 26.2% of PCF patients; perhaps alcohol mitigates TB symptoms and pushes people away from social institutions including the health care system. ACF decreases time delay: PCF patients had a median PD of 4 weeks (IQR 1–8 weeks), only 23% of them were seen during first week from symptoms onset, while most ACF patients were seen within 1 week.

One third (34.7%) of patients had no symptoms during the first visit to the health care system. They contacted doctors because of such “obligatory” demands as a required annual medical examination, or X-ray examination or because of contact with a TB patient (Table I). Moreover, 24.6% of ACF patients reported symptoms but did not ask for medical help before screening. It seems that patients denied TB symptoms in daily life, which agreed with other studies that found only a deadly threat is a motivator for contacting a health system (55). One fifth of the patients (21.0%) came to a hospital as an emergency admission. The fact that 40% had sputum from 2 till 62 weeks before the visit to a doctor showed that they was perceived sputum production as a “nothing unusual” situation. According to other research, doctors often miss respiratory symptoms because they are “normal” for some patients (12). Russian researchers described that often many data from anamnesis, clinical data and examination results were not used by doctors for diagnostic decision making (54). This was not supported by our findings, because most of the patients had HSD less than 2 weeks. We can say that PD was more important in our setting (Tables II and III). Our findings showed that about half of the patients (45.8%) visited the health system 2 weeks from symptom onset and would not be picked up by the PCF strategy.

Some authors described stigma as an important barrier to TB and other infectious disease diagnosis (56, 57). This was not supported by our previous study (unpublished data). Socially less vulnerable people who used self-treatment showed a longer time of PD (Table III). This contradicts numerous studies that reported an association between patient delay and low education and inadequate housing (40, 44, 52, 53). Patients who had attained a higher educational level also had a longer time of PD (Tables III and IV). A possible explanation is that sick persons without housing cannot live in a harsh climate and reported to the health system earlier. These persons were alcohol abusers and had a shorter PD than alcohol users (Table II). Patients with more education often have comfortable housing, so they may have a longer time of PD. Moreover, they use self-treatment, which increases PD (40, 44, 52, 53). Additionally, there were no differences among ACF patients because ACF strategies do not consider these factors. HSDs were less than 2 weeks for the majority of the patients (77.5%). It seems that the main reason for DD is a low level of patient attention to their own conditions. If a patient reaches a health service and shows symptoms, he/she will be diagnosed in a short time.

Gender can increase or decrease DD time depending on country customs related to stigma (40, 44, 52, 53). We found longer male PD in passive case finding (Table IV), which was supported by qualitative data stating that men were more likely to have patient delays than women, who were more likely to have health system delay (unpublished data).

## Conclusion

Patients diagnosed through ACF tended to under-report their TB symptoms and showed low attention to their own health. However, ACF allowed TB patients to be revealed earlier than PCF and this was also the case for alcohol abusing patients. In risk groups, ACF is more effective than PCF. PCF systems should be supplemented with ACF strategies for risk groups (homeless, prisoners, household contact with TB patients, HIV patients).
